# Dissertation on the Signs of Certain Death, Previously to Putrefaction

**Published:** 1837-07

**Authors:** 


Art. VII.—*Dissertationis de Signis Mortem hominis absolutam ante Putredinis accessum indicantibus particula prior et posterior.* Auctore A. G. Sommer, m.d.—*Hafniæ*, 1833. 8vo.

*Dissertation on the Signs of certain Death, previously to Putrefaction.* By A. G. Sommer, m.d.—*Copenhagen*, 1833. Two Parts. 8vo. pp. 277.

The knowledge of the signs of real death is certainly of the greatest importance, not only to the medical man, but to everybody, and authors of late years have done right in endeavouring to ascertain such as might be relied on in all cases. From want of such knowledge, the earlier writers and chroniclers relate many instances of men that were buried as dead, and yet only were apparently so. Some of these are not wholly to be doubted, and the *possibility* of such an accident occurring must at any rate be allowed, as long as the study of the signs of real and apparent death was neglected. Even now, when so much has been written and observed concerning this subject, it is so far from being fully illustrated, that the best-informed will be still liable to mistakes. On that account we welcome every more exact investigation of those signs of death which have been delivered to us as guides in doubtful cases; and the author of the two small volumes now before us has therefore, from the mere *choice* of his subject, a just claim upon our thanks.

The signs of real death are most conveniently divided (1) into those which take place *before* putrefaction, and which precede it, and (2) into those which accompany putrefaction. Several authors have asserted that only putrefaction itself, and the other phenomena connected with it, are to be considered as *true* signs of absolute death, and to put it beyond all question; and that those signs, on the contrary, that belong to the first category are never to be trusted. Yet it is clear of what importance it must be to the medical man to be able to form an opinion as to real or apparent death from the signs before the commencement of putrefaction: for instance, in camps, during epidemic contagious diseases—when, to prevent contagion, the dead must soon be removed, &c.

The positive signs of real death before putrefaction are derived either (1) from the quality of the causes that might have occasioned them; or (2) from the evident want of all the functions that indicate life, even in its slightest manifestation; or (3) from the inefficacy of the surgical or other means used during a sufficient period to recall life. It is particularly the signs of the second class that often create a doubt.

The diagnostic signs that precede real putrefaction are in general stated to be the following:—(1.) The body is quite cold. (2.) A general stiffness of all the parts (*rigor cadaverosus,*) has arisen. (3.) The circulation of the blood has ceased; neither pulsation of the arteries nor of the heart can be perceived. (4.) Blue and red spots and streaks are observed, particularly on the posterior parts of the body. (5.) Respiration has quite ceased. (6.) No mobility of the parts exists, but elasticity and contractility are gone; and (7.) No remains of sensation can be detected.

These principal signs of real death, as quoted by authors, Dr. Sommer resolved to examine more closely; and, though his work was merely written as an inaugural dissertation, for the purpose of obtaining the degree of doctor of medicine; yet it is so very superior to the generality of dissertations of this class, that we feel it incumbent on us to call the attention of the profession to it. It contains much interesting and much valuable matter; some new, some not new but renovated and strengthened by new experiments. Of all the signs of death before putrefaction, Dr. Sommer lays the greatest stress upon the *rigor cadaverosus* as the surest test. But our principal object, on the present occasion is to direct the attention of the reader to a phenomenon not hitherto noticed, which, according to the experiments and investigations of the author, only takes place in the really dead body, and in certain cases may become of importance as a sign of death. Dr. Sommer is fully entitled to the credit of having been the first to observe, or at least to communicate to the profession, this new sign of death: it is ***a blackish hue of the sclerotica of the eyes.***

"Most of those who die," says Dr. Sommer, "have the eyelids half opened; when, immediately after death, I examined the body, I observed a natural, equable whiteness of the sclerotica; but after the expiration of one, two, or three hours, the colour of this membrane was observed to be *yellow* in that part of it *which had been exposed*

*to the air and to the light* between the opened eyelids; while that part of it that had been covered by the eyelids yet possessed its natural white hue. In a man, who had died with the eyes open, the sclerotica acquired a *blackish blue colour.*"

This excited the attention of the author, and he instituted the following experiment upon the dead body of an individual who had died from inflammation of the intestines, as a consequence of incarcerated hernia. Of the right eye he fastened the eyelids carefully down, by means of sticking-plaster and a bandage, in order to exclude entirely the action of light; but of the left eye he drew the upper eyelid up, and kept it in this state by means of sticking-plaster, so that the whole cornea and a great part of the sclerotica remained exposed to light. After the expiration of five hours, he then examined both eyes, and found the sclerotica in the *right* (closed) eye quite white, but that in the *left* (opened) just as far as it had been exposed to the light, of a yellow hue, which near the cornea changed to *black.* The cornea of the *left* eye was also *opaque*, but that of the *right*, *transparent.*

The same phenomenon took place in all the dead bodies on which this experiment was instituted, provided it was made during the first hours after death, before the eyeball had collapsed. The greater the natural distension or fulness of the eyeball, the more distinct is the blackish blueness; but afterwards, when the eyelids are brought together, and particularly when the eyes become depressed, it diminishes very much, or disappears altogether. This colour arises often very soon after death, forming then usually a triangle, with its base directed towards the cornea (where the colour is most marked), and with its point situated at the external angle of the eye.

Sometimes also in collapsed eyes this change of colour is produced, but never to the same degree as in those still retaining the natural turgescence. It is likewise found that this change of colour is greater and more equal in the sclerotica, at the external angle of the eye, than on the other parts of it; in these it is slighter and dispersed in spots. When the eyelids again are closed, the hue on the next day becomes so altered, that it is only *yellow*, a hue, which is likewise sometimes met with in collapsed eyes that have not been kept open.

The author has never observed such a remarkable change of hue in individuals still living; only in some typhous and phthisical patients, during the agonies of death, bluish spots have been formed at the external and nether part of the sclerotica, which, after death, assumed the afore-mentioned hue. Something similar has been remarked in patients with the Asiatic cholera, but in these cases *beneath* the cornea.

The author ascribes this phenomenon, which he at first was inclined to attribute to the exudation of the black pigment through the choroid, (but which opinion was disproved by an anatomical dissection,) to the exsiccation of the sclerotica and the *adnata.* The sclerotic is exsiccated in the same manner as other fibrous membranes after death, by the access of air, and becomes transparent, so that the choroid and the black pigment shine through it; only that part, that is exposed to the air changes its hue. This conjecture is supported by the result of an experiment which Dr. Sommer instituted on an individual recently after death, whose eyes still preserved the natural tension. He drew the lids up, exposed the eyes to the air, and kept one eye constantly moistened with water, but the other eye dry. After the expiration of two hours, he found the sclerotica of the moistened eye, as white as at the moment of death, but that of the other, very blue. That the change of colour is not so marked in collapsed eyes, is undoubtedly owing to a partial dissolution and beginning decomposition of the black pigment.

That the blackish hue is most distinct *near the cornea*, must be explained by the fact of the black pigment lying in thicker layers at this part of the eye; probably also from greater thinness of the sclerotica at this part. Yet the change of hue does not extend itself into the margins of the cornea; there is always as much as a line in breadth around the cornea, where the black hue either is not observed at all, or is very inconsiderable; and this may be explained by the presence of the ciliary ligament in this place. The hue is perhaps not perfectly black, because the colour of the tela cellulosa in the sclerotic and choroid is yellow, and the sclerotic itself white. That the blueness of the eyes is diminished, or fades entirely away after the closing of the eyelids, may arise from the exsudations which take place in all dead bodies, and by which the eyeball is moistened.

The author does not claim the name of a perfectly sure sign of death for the phenomenon above described, and modestly requests his medical brethren to institute experiments themselves with regard to it; if it should be confirmed as a certain sign, it will be admitted to be of the more importance, as it shows itself quickly after death, and is easily observed.

